# Time trends in antibiotic consumption in the elderly: Ten-year follow-up of the Spanish National Health Survey and the European Health Interview Survey for Spain (2003–2014)

**DOI:** 10.1371/journal.pone.0185869

**Published:** 2017-11-29

**Authors:** Domingo Palacios-Ceña, Valentín Hernández-Barrera, Isabel Jiménez-Trujillo, Ramón Serrano-Urrea, César Fernández-de-las-Peñas, Pilar Carrasco-Garrido

**Affiliations:** 1 Department of Physiotherapy, Occupational Therapy, Rehabilitation and Physical Medicine. ITPSE Research Group. Universidad Rey Juan Carlos. Alcorcón, Madrid. Spain; 2 Medicine and Surgery, Psychology, Preventive Medicine and Public Health, Medical Microbiology and Immunology and Nursing Department. Universidad Rey Juan Carlos. Alcorcón, Madrid; 3 Medicine and Surgery, Psychology, Preventive Medicine and Public Health, Medical Microbiology and Immunology and Nursing Department. Universidad Rey Juan Carlos. Alcorcón, Madrid; 4 Department of Mathematics. Faculty of Computer Science Engineering. University of Castilla-La Mancha. Albacete, Castilla la Mancha. Spain; 5 Department of Physiotherapy, Occupational Therapy, Rehabilitation and Physical Medicine. ITPSE Research Group. Universidad Rey Juan Carlos. Alcorcón, Madrid. Spain; 6 Medicine and Surgery, Psychology, Preventive Medicine and Public Health, Medical Microbiology and Immunology and Nursing Department. ITPSE Research Group. Universidad Rey Juan Carlos. Alcorcón, Madrid. Spain; Universidade Federal de Sao Paulo, BRAZIL

## Abstract

**Background:**

The purposes of this study were: firstly, to estimate time trends in the prevalence of prescription antibiotic consumption between 2003 and 2014; secondly, to identify the factors associated with the probability of consuming antibiotics during this period in elderly persons in Spain.

**Methods:**

We analyzed data collected from the Spanish National Health Survey in 2003 (n = 21,650), 2006 (n = 29,478), and 2012 (n = 20,007) and from the European Health Interview Survey for Spain in 2009 (n = 22,188) and 2014 (n = 22,842). Antibiotic consumption was the dependent variable. We also analyzed sociodemographic features, self-perceived health status, lifestyle habits, comorbid diseases, and disabilities using logistic regression models.

**Results:**

The prevalence of antibiotic consumption increased from 2003 to 2014 in both sexes. The variables that predicted antibiotic consumption (men; women) were secondary education (OR 1.38; OR 1.31), visits to a general practitioner (OR 2.05; OR 2.15), hospitalization (OR 1.91; OR 1.83), therapy with > 4 non-antibiotic drugs (OR 3.36; OR 5.84), instrumental activities of daily living (OR 1.50; OR 1.24), and activities of daily living (OR 1.39; OR 1.35). In contrast, age > 85 years was associated with low antibiotic consumption in both men (OR 0.81) and women (OR 0.88).

**Conclusions:**

The prevalence of antibiotic prescription has increased in Spain in the last decade. Our study identified several factors that appear to affect antibiotic consumption in elderly persons, with potential implications for healthcare providers.

## Introduction

Antibiotics reduce the burden of common infectious diseases and usually play an essential role in many medical interventions because of their therapeutic benefits and their impact on public health throughout the world [1]. However, patterns of use of antibiotics have become the main cause of bacterial resistance [2,3]. Antibiotic consumption has increased in the EU during the last 10 years, with variations between countries within the EU and countries from outside [4,5]. According to the European Surveillance of Antimicrobial Consumption Network (ESAC-Net), the weighted mean consumption of antibiotics by the population of the EU/EEA increased significantly between 2009 and 2013, particularly in Belgium, Ireland, Latvia, Norway, Spain, and the United Kingdom. Spain has one of the highest rates of consumption, which reached 24.2 DDD/1000 inhabitants/day in 2013, i.e., higher than the mean 22.4 DDD/1000 inhabitants/day for the EU during the same year [6].

Similarly, previous studies have shown over prescription of these drugs outside hospitals which is associated with more frequent use of healthcare services for treatment of specific conditions [7,8] and a greater expectation of receiving antibiotics by the patients [5]. Also, previous studies reported sex and age differences in the use of antibiotic prescription [9,10]. In this context, individuals aged >65 years old in Spain consume more than 30% of drugs prescribed daily for the treatment of chronic diseases [11]. Prescription of antibiotics among elderly patients has been reported to be very frequent, with a prevalence of consumption ranging between 1.2% [12] and 9.4% [13] in nursing homes and up to 48% among elderly persons not living in institutions [5].

The objectives of the present study were: a) to analyze time trends in the prevalence of consumption of prescription antibiotics among elderly persons in Spain between 2003 and 2014; and, b) to identify the sociodemographic features, self-rated health status, comorbidity, lifestyle habits, and disabilities associated with antibiotic consumption during this period in elderly persons in Spain.

## Materials and methods

### Data source

We conducted a nationwide, descriptive, cross-sectional epidemiological study on the consumption of antibiotics by persons aged ≥65 years old in Spain. We used individualized secondary data drawn from the 2003, 2006, and 2012 Spanish National Health Surveys (SNHS) [14–16] and the 2009 and 2014 European Health Interview Surveys for Spain (EHIS) [17,18]. The SNHS is an ongoing survey that collects data by home-based personal interview in order to examine a national representative sample of the non-institutionalized population residing in main family dwellings (households) in Spain. The surveys use multistage cluster sampling, with proportional random selection of primary and secondary sampling units (towns and sections, respectively); the final units (individuals) are selected using random routes and gender- and age-based quotas. Surveyors were trained in basic communication skills, procedures, and administration of the questionnaire. Details of SNHS methodology are reported elsewhere [14–16].

The 2003 survey included 21,650 adults of both sexes interviewed between April 2003 and March 2004, the 2006 survey included 29,478 adults of both sexes interviewed between June 2006 and June 2007, and the 2012 survey included 20,007 persons interviewed from July 2011 to June 2012. Data from the years 2009 and 2014 were obtained from the EHIS, which was proposed by the European Commission to EU Member States and conducted by the Spanish National Statistics Institute under the aegis of the Spanish Ministry of Health, Social Affairs, and Equality (SMHSAE). The methods of the EHIS are the same than those used in the SNHS. The data collection period ranged from April 2009 to March 2010, and a sample of 22,188 subjects aged 16 or over was selected. The 2014 survey included 22,842 adults of both sexes interviewed between January 2014 and January 2015. A detailed description of EHIS methodology can be found elsewhere [17,18].

### Variables

For the purposes of the current study, we included answers from adults of both sexes aged ≥65 years from all the surveys. The variables included can be compared because the same questions were asked in all 5 surveys.

The information to create the dependent variables was obtained from the answers "yes" or "no" to the question “Have you taken antibiotics in the last 2 weeks?” Respondents who answered “yes” were then asked the question “Were they prescribed for you by a doctor?” Those who answered yes to the first question, regardless of the answer to the second one, were considered consumers of antibiotics.

The independent variables were the primary sociodemographic characteristics of the population, namely, age, marital status, and educational level. To identify subjects with an associated chronic condition, we used the self-reported affirmative answer to the presence of any of the following physician-diagnosed diseases (categorized as none, 1–2, ≥3): arterial hypertension, hypercholesterolemia, respiratory disease (asthma and chronic bronchitis), heart disease, diabetes, and cancer. The variables related to lifestyle and health profile used in the study were current smoking (yes/no), consumption of alcoholic beverages within the 2 weeks prior to the survey (both defined as dichotomous variables), body mass index, and leisure time physical activity (moderate, light, or none). Self-perceived health was analyzed as a dichotomous variable (very good and good/fair or poor and very poor).

In order to assess the use of healthcare resources, respondents were asked about visits to the primary care physician (in the preceding 4 weeks [yes/no]), hospitalization (in the previous 12 months [yes/no]), and visits to the emergency department (in the previous 12 months [yes/no]). They were also asked if they had taken any type of non-antibiotic medication in the previous 2 weeks (categorized as none, 1–3, ≥4).

Finally, information regarding functional disability was obtained from questions targeting distinct physical tasks in 2 functional domains that had been validated and used in previous studies [19,20], as follows: (1) activities of daily living (ADL) (i.e., bathing or showering, dressing and undressing, feeding oneself, getting in and out of a bed or chair, and using the toilet); (2) instrumental activities of daily living (IADL) (i.e., preparing meals, taking care of finances and everyday administrative tasks, doing light housework, managing medication, and use of the telephone). Participants were defined as being functionally impaired in a particular domain if they answered in at least one of the specific tasks in these domains with any of the following: “Yes, I can do it but with some difficulty”, “Yes, I can do it with a lot of difficulty”, or “I cannot do it by myself”.

### Statistical analysis

We calculated the prevalence of total antibiotics consumption for each of the 5 surveys according to the study variables. All data analyses were performed separately for women and men. Pearson’s χ^2^ test was used for the bivariate comparison of proportions, and statistical significance was set at p < 0.05 (2-tailed).

To estimate the independent effect of each of the variables on the consumption of antibiotics, we also obtained the corresponding adjusted odds ratio (AOR) by means of multivariate logistic regression analysis. All variables with a significant association in the bivariate analysis were included in the multivariate analysis, along with those variables that were considered relevant in the scientific literature. Two models were generated to identify factors associated with the overall consumption of antibiotics in men and women.

Estimates were made by incorporating the sampling weights and using the “svy” (survey command) functions of STATA (STATA Corp, College Station, Texas, USA), which enabled us to incorporate the sampling design into all our statistical calculations (descriptive, χ^2^ test, logistic regression). Statistical significance was set at a 2-tailed α<0.05.

### Ethical statements

In accordance with Spanish legislation, there is no need for Ethics Committee approval, since the database was obtained from the webpage of SMHSAE, where it is publicly available. The data were analyzed anonymously.

## Results

The study population included 12,294 men (42.91%) and 20,117 women (57.09%), of whom 52.36% were aged 65–74 years, 36.42% were aged 75–84 years, and 11.22% were aged ≥85 years.

Consumption of antibiotics increased significantly during 2003–2014 in both sexes, 5.6% in men (p = 0.002) and 6.21% in women (p = 0.001). By age, consumption increased in women aged 65–74 years and 75–84 years. During the years 2009 and 2012, statistically significant values were recorded for the age ranges 65–74 years and ≥ 85 years, respectively (p<0.05) in both sexes (Table 1).

**Table 1:** Prevalence of antibiotic consumption in Spanish elderly, according to sex and age groups. Spanish National Health Survey (SNHS) 2003–2006, 2012 and European Health Interview Survey (EHIS) 2009 and 2014 for Spain.

		2003	2006	2009	2012	2014	p-value
Age	Sex	n (%)	n (%)	n (%)	n (%)	n (%)	
**65–74** ^**a**^	**Male**	39(2.97)	78(5.35)	39(3.23)	41(3.59)	70(5.07)	0.004
	**Female**	74(3.69)	134(5.42)	85(5.18)	80(5.04)	103(5.9)	0.018
**75–84**	**Male**	25(3.08)	60(5.37)	34(3.82)	52(6.24)	63(6.76)	0.233
	**Female**	47(3.28)	112(5.49)	56(3.65)	80(5.28)	97(6.51)	0.000
**≥ 85** ^**b**^	**Male**	7(3.98)	16(6.75)	8(3.45)	20(8.13)	14(4.49)	0.831
	**Female**	20(5.09)	26(5.1)	20(3.85)	25(4.39)	42(6.36)	0.269
**Total**	**Male**	71(3.08)	154(5.47)	81(3.48)	113(5.08)	147(5.6)	0.002
	**Female**	141(3.68)	272(5.42)	161(4.36)	185(5.04)	242(6.21)	0.001

^a^ Statistically significant differences (p < 0.05) EHIS 2009

^b^ Statistically significant differences (p < 0.05) SNHS 2012.

Data consumption in men, according to sociodemographic characteristics, lifestyle, and health-related study variables are shown in S1 Table. Consumption increased during 2003–2014 (p = 0.002), especially in men aged 65–74 years (p = 0.004). Consumption in elderly persons increased during 2003–2014 with respect to educational level, marital status, income, lifestyle, visits to health centers, polymedication, and disability.

Chronic diseases were significantly associated with consumption of antibiotics during each year of the study period (p<0.05), although no significant increases were recorded.

S2 Table shows the prevalence of antibiotics consumption according to sociodemographic variables and health in women. During 2003–2014, consumption increased among women (p = 0.001) aged 65–74 years (p = 0.018) and 75–84 years (p = 0.002). Consumption increased in both sexes and with respect to secondary education, marital status and income, lifestyle, visits to health centers, and disability in ADL. Unlike in men, consumption increased in women who did not have difficulties in performing ADL (p = 0.025) and who did not consume medications other than antibiotics (p = 0.006).

The results of the multivariate analysis are shown in Table 2. Male sex tended to be a predictor of antibiotic consumption (AOR, 1.17; 95%CI, 1.02–1.33).

**Table 2:** Factors associated with consumption of antibiotics by prescription in elderly persons aged ≥65 years in Spain. Spanish National Health Survey 2003–2006, 2012 and European Health Interview Survey 2009 and 2014 for Spain.

		Male	Female
		AOR, 95%CI	AOR, 95%CI
**Age**	**65–74**	1	1
	**75–84**	1.01(0.80–1.26)	0.78(0.65–0.94)
	**≥ 85**	0.81(0.57–1.17)	0.88(0.67–1.14)
**Educational level**	**No formal education**	1	1
	**Primary education**	1.11(0.87–1.42)	0.89(0.75–1.06)
	**Secondary education**	1.38(1.03–1.85)	1.31(1.01–1.69)
**Medical consultation**	**No**	1	1
	**Yes**	2.05(1.60–2.63)	2.15(1.76–2.61)
**Hospitalization in preceding 12 months**	**No**	1	1
	**Yes**	1.91(1.53–2.39)	1.83(1.52–2.21)
**Number of non-antibiotic drug**	**None**	1	1
	**1–3**	1.28(0.82–1.99)	2.13(1.33–3.41)
	**≥4**	3.36(2.15–5.23)	5.84(3.75–9.38)
**Instrumental Daily Living Activities**	**No**	1	1
	**Yes**	1.50(1.16–1.95)	1.24(1.01–1.51)
**Daily Living Activities**	**No**	1	1
	**Yes**	1.39(1.07–1.84)	1.35(1.09–1.66)
**SNHS and EHIS**	**2003**	1	1
	**2006**	1.57(1.08–2.28)	1.37(1.05–1.80)
	**2009**	0.98(0.65–1.48)	1.28(0.95–1.72)
	**2012**	1.25(0.84–1.84)	1.29(0.97–1.78)
	**2014**	1.46(1.01–2.11)	1.66(1.27–2.18)

AOR: Odds Ratio; CI: Confidence Interval; SNHS: Spanish National Health Survey; EHIS: European Health Interview Survey.

When the consumption pattern among women was analyzed, the variables that were independently and significantly associated with a greater probability of antibiotic use were age 75–84 years (AOR, 0.78; 95%CI, 0.65–0.94), educational level, visits to the general practitioner (GP), hospitalization, polymedication with ≥ 4 nonantibiotic drugs (AOR, 5.84; 95% CI, 3.75–9.38), and disability (ADL and IADL).

As for elderly men, the health profile analysis showed that educational level, visits to a GP, taking 4 or more non-antibiotic medications (AOR, 3.36; 95% CI, 2.15–5.23), ADL, and IADL were all significantly associated with more frequent consumption of antibiotics.

To assess the time trend in the consumption of antibiotic during the period 2003–2014 and after adjusting for differences in socioeconomic data and health status, antibiotic consumption increased by 66% between 2003 and 2014 in elderly women (AOR, 1.66; 95%CI, 1.27–2.18), whereas antibiotic consumption among Spanish elderly men increased by 46% during the study period (AOR, 1.46; 95%CI, 1.01–2.11).

## Discussion

Our study revealed an increase in antibiotic consumption from 2003 to 2014 in elderly persons in Spain. We found that variables associated with higher consumption were secondary education, visits to a GP, hospitalization, taking ≥ 4 non-antibiotic drugs, and difficulty in performing IADL and ADL. In contrast, age ≥ 85 years was associated with low consumption in both sexes.

Our study supports previous reports of increases in consumption of antibiotics in elderly [3,21]. Increased antibiotic prescription fill rates in the elderly may be explained by a change in patient behavior [10,22], physician education [10,23], seasonal differences in antibiotic use [21], antibiotic price reimbursement [3,22], type of infection [3,24], choice of antibiotics based on national policies [22], and application of antimicrobial treatment to prevent complications and loss of autonomy [25]. Further, the pressure exerted by the pharmaceutical industry on GPs plays an important part in the choice of antibiotic [26].

We observed a variation in prevalence in 2009, probably because the purchasing power of elderly persons decreased during the economic recession and prescriptions were more closely controlled, thus potentially leading to misuse of medication [27,28].

Our results showed that antibiotic consumption increased in both sexes and are consistent with previous studies [9,10,22,26,29], which reporting that the prevalence of antimicrobial use changed markedly between genders.

In some cases, gender differences can be explained by the greater frequency of symptomatic infections of the urinary tract in women than in men and the over-use of primary healthcare services by women [20,30].

Previous studies [9,29,31,32] reported higher rates of consumption in advanced age. In Holland, Haeseker et al [32] showed that antibiotic prescriptions increased in age >80 years during 2000–2009 (p<0.001). In contrast, our results showed that age ≥ 85 years old was associated with a decreased probability of antibiotic consumption. Although, the reason why this decreased occurred cannot be inferred by the nature of the study design, a negative OR value may be explained in part by efforts to limit antibiotic prescription in the elderly in order to protect them [33]. Thus, anticipating drug interactions by minimizing the unnecessary use of antibiotics may help prevent adverse events [32], antibiotic resistance [24,34], and secondary infections (i.e. *Clostridium difficile*)[34].

Low educational level [35] was also significantly associated with antibiotic consumption. In a study conducted in São Paulo (Brazil), Kliemann et al [36] reported how antibiotic consumption was more frequent in larger populations with higher educational levels. It is possible that education acts differently in developing countries, where it may improve access to medical treatment, than in developed countries, where access to medical treatment may not depend on educational levels, despite increasing knowledge about health patterns and correct antibiotic use. Furthermore, consumption could be determined by patients’ misconceptions about antibiotics [23], inadequate knowledge about appropriate use of antibiotics [22], and cultural background [37].

Previous studies have shown that visiting a GP was associated with increased consumption of antibiotics by the elderly [7,10,31,32]. The reason why GPs prescribe antibiotics in response to patient demands is to avoid patient dissatisfaction with the visit [7,10,23]. Also, the prescription could validate the seriousness of the visit, thus reassuring the patient that he or she was right to come to the GP and seek treatment [5].

Hospitalization is also a risk factor for consumption of antibiotics in terms of the admitting complaint, disease burden, iatrogenic infections associated with urinary catheters, and emergence of multidrug-resistant infections [24,31].

In general, although concomitant use of multiple drugs is very common among elderly persons in developed countries, data on the prevalence of polypharmacy in the literature also vary widely between studies. This practice can mask symptoms of infection (fever, cough, pain), thus hampering diagnosis and necessitating prescription of broad-spectrum antimicrobial drugs to provide full antibiotic coverage [24]. Paradoxically, use of antibiotics together with other drugs can lead to type A adverse reactions (dose-related) or type B reactions (immunologic drug reactions, drug intolerance) [24,38]. Thus, Haeseker et al [32] reported that the highest risk group to develop adverse drug events is aged over 80 years with multiple co-morbidity and co-medication.

Our results also showed that functional disability increased the probability of antibiotic consumption. There are several explanations for this finding. Disability is associated with increased severity of infection in persons aged > 80 years and could be an atypical manifestation of infection in elderly persons (urinary tract infection, pneumonia) [38]. In addition, the loss of ADL (eating, bathing, transferring) could increase the risk of infection. Moreover disability is a predictor of mortality and a criterion for early use of antibiotics in elderly persons with severe infections [38] and has been associated with the emergence of multidrug-resistant infections [38,39].

### Limitations

First, we used a self-reported measure of antibiotic consumption, which may have limited the assessment of antibiotic prescription. Nevertheless, even though individuals can overestimate or underestimate their antibiotic consumption, surveys are extremely useful for investigating patterns, frequencies, and longitudinal trends of antibiotic consumption [40]. Second, we did not consider institutionalized or hospitalized population, rather than identifying specific active pharmaceutical ingredients, the SNHS and the EHIS, identify groups of medicines for specific diseases, or disorders. Third, the study design did not enable us to determine a cause and effect relationship owing to the lack of longitudinal follow-up of the same population. Nevertheless, the use of a national and European population-based survey makes it possible to include representative national sample sizes. Despite these limitations, our study provides additional insight into demographic aspects (age, sex) of antibiotic consumption in older adults for whom there is little information at the population level, particularly in Spain. Lastly, given that the initial response rates to the SNHS and EHIS were low (61% to 65%) [19–23], the possibility of non-response bias must be taken into consideration.

## Conclusions

The current study found an increase in antibiotic consumption in Spain between 2003 and 2014. Factors associated with higher consumption were secondary education, visits to a GP, hospitalization, taking ≥ 4 non-antibiotic drugs, and poor functional status. Our results have clear implications for health services in Spain. At national level, health services and hospitals should develop joint programs focused on appropriate antibiotic prescription and development of programs to monitor consumption in elderly people. Further, the creation of programs to monitor antimicrobial use, and identification of factors associated to increase of consumption should be carried out. At a community level, in primary health care, health care professionals could increase elderly education to prevent inappropriate antibiotic consumption, and identify factors associated to increase GP visits.

## Supporting information

S1 Table Prevalence of consumption of prescription antibiotics in men. (DOC)

S2 Table Prevalence of consumption of prescription antibiotics in women. (DOC)

## References

[1] Van Boeckel TP, Gandra S, Ashok A, Caudron Q, Grenfell BT, Levin SA, et al Global antibiotic consumption 2000 to 2010: an analysis of national pharmaceutical sales data. Lancet Infect Dis. 2014;14(8):742–50. doi: 10.1016/S1473-3099(14)70780-7 25022435 10.1016/S1473-3099(14)70780-7

[2] World Health Organization. Antimicrobial resistance: global report on surveillance. Available from: http://www.who.int/drugresistance/documents/surveillancereport/en/. Published 2014. Accessed July 18, 2017.

[3] Shapiro DJ, Hicks LA, Pavia AT, Hersh AL. Antibiotic prescribing for adults in ambulatory care in the USA, 2007–09. J Antimicrob Chemother 2014; 69: 234–40. doi: 10.1093/jac/dkt301 23887867 10.1093/jac/dkt301

[4] Rún Sigurðardóttir N, Nielsen AB, Munck A, Bjerrum L. Appropriateness of antibiotic prescribing for upper respiratory tract infections in general practice: Comparison between Denmark and Iceland. Scand J Prim Health Care. 2015;33(4):269–74. doi: 10.3109/02813432.2015.1114349 26683287 10.3109/02813432.2015.1114349 PMC4750736

[5] Stearns CR, Gonzales R, Camargo CA Jr, Maselli J, Metlay JP. Antibiotic prescriptions are associated with increased patient satisfaction with emergency department visits for acute respiratory tract infections. Acad Emerg Med. 2009;16(10):934–41. doi: 10.1111/j.1553-2712.2009.00522.x 19799568 10.1111/j.1553-2712.2009.00522.x

[6] European Centre for Disease Prevention and Control. European Surveillance of Antimicrobial Consumption Network (ESAC-Net). Summary of the latest data on antibiotic consumption in the European Union. Available from: http://ecdc.europa.eu/en/eaad/antibiotics-get-informed/antibiotics-resistance-consumption/Documents/antibiotics-consumption-EU-data-2014.pdf.

[7] Dekker AR, Verheij TJ, van der Velden AW. Inappropriate antibiotic prescription for respiratory tract indications: most prominent in adult patients. Fam Pract. 2015;32(4):401–7. doi: 10.1093/fampra/cmv019 25911505 10.1093/fampra/cmv019

[8] Schroeck JL, Ruh CA, Sellick JA Jr, Ott MC, Mattappallil A, Mergenhagen KA. Factors associated with antibiotic misuse in outpatient treatment for upper respiratory tract infections. Antimicrob Agents Chemother. 2015;59(7):3848–52. doi: 10.1128/AAC.00652-15 25870064 10.1128/AAC.00652-15 PMC4468652

[9] Lallana-Alvarez MJ, Feja-Solana C, Armesto-Gómez J, Bjerrum L, Rabanaque-Hernández MJ. Outpatient antibiotic prescription in Aragón and the differences by gender and age. Enferm Infecc Microbiol Clin. 2012;30(10):591–6. doi: 10.1016/j.eimc.2012.03.004 22534155 10.1016/j.eimc.2012.03.004

[10] Bagger K, Nielsen AB, Siersma V, Bjerrum L. Inappropriate antibiotic prescribing and demand for antibiotics in patients with upper respiratory tract infections is hardly different in female versus male patients as seen in primary care. Eur J Gen Pract. 2015;21(2):118–23. doi: 10.3109/13814788.2014.1001361 25712495 10.3109/13814788.2014.1001361

[11] Lago JA, Viveros JE, Heredia J. [Pharmaceutical expenditure in Spain. International developments, national situation and expenditure control]. Escuela de Administración de Empresas Business School Available at: http://www.redaccionmedica.com/contenido/images/eae gasto farmaceutico.pdf. Published 2012. Accessed July 18, 2017.

[12] Zimmerman S, Sloane PD, Bertrand R, Olsho LE, Beeber A, Kistler C. Successfully reducing antibiotic prescribing in nursing homes. J Am Geriatr Soc. 2014;62(5):907–12. doi: 10.1111/jgs.12784 24697789 10.1111/jgs.12784

[13] McClean P, Hughes C, Tunney M, Goossens H, Jans B; European Surveillance of Antimicrobial Consumption (ESAC) Nursing Home Project Group. Antimicrobial prescribing in European nursing homes. J Antimicrob Chemother. 2011;66(7):1609–16. doi: 10.1093/jac/dkr183 21596722 10.1093/jac/dkr183

[14] Instituto Nacional de Estadística. Spanish Nacional Health Survey 2003. Available from: http://www.ine.es/dyngs/INEbase/es/operacion.htm?c=Estadistica_C&cid=1254736176783&menu=resultados&secc=1254736194726&idp=1254735573175.

[15] Instituto Nacional de Estadística. Spanish National Health Survey 2006. Available from: http://www.ine.es/dyngs/INEbase/es/operacion.htm?c=Estadistica_C&cid=1254736176783&menu=resultados&secc=1254736194724&idp=1254735573175.

[16] Instituto Nacional de Estadística. Spanish National Health Survey 2012. Available from: http://www.ine.es/dyngs/INEbase/es/operacion.htm?c=Estadistica_C&cid=1254736176783&menu=metodologia&idp=1254735573175.

[17] Instituto Nacional de Estadística. European Health Interview Survey for Spain 2009. Available from: http://www.ine.es/dyngs/INEbase/es/operacion.htm?c=Estadistica_C&cid=1254736176784&menu=resultados&secc=1254736195297&idp=1254735573175.

[18] Instituto Nacional de Estadística. European Health Interview Survey for Spain 2014. Available from: http://www.ine.es/dyngs/INEbase/es/operacion.htm?c=Estadistica_C&cid=1254736176784&menu=resultados&secc=1254736194728&idp=1254735573175.

[19] Palacios-Ceña D, Jiménez-García R, Hernández-Barrera V, Alonso-Blanco C, Carrasco-Garrido P, Fernández-de-Las-Peñas C. Has the prevalence of disability increased over the last decade (2000–2007), in elderly people? A Spanish population-based survey. J Am Med Dir Assoc. 2012; 13(2):136–42. doi: 10.1016/j.jamda.2010.05.007 21450186 10.1016/j.jamda.2010.05.007

[20] Palacios-Ceña D, Hernández-Barrera V, Jiménez-García R, Valle-Martín B, Fernández-de-las-Peñas C, Carrasco-Garrido P. Has the prevalence of health care services use increased over the last decade (2001–2009) in elderly people? A Spanish population-based survey. Maturitas. 2013;76(4):326–33. doi: 10.1016/j.maturitas.2013.07.016 23972333 10.1016/j.maturitas.2013.07.016

[21] Zhang Y, Steinman MA, Kaplan CM. Geographic variation in outpatient antibiotic prescribing among older adults. Arch Intern Med. 2012;172(19):1465–71. doi: 10.1001/archinternmed.2012.3717 23007171 10.1001/archinternmed.2012.3717 PMC3530642

[22] Kourlaba G, Gkrania-Klotsas E, Kourkouni E, Mavrogeorgos G, Zaoutis TE. Antibiotic prescribing and expenditures in outpatient adults in Greece, 2010 to 2013: evidence from real-world practice. Euro Surveill. 2016;21(26). 10.2807/1560-7917.ES.2016.21.26.30266 27390126

[23] Pan DS, Huang JH, Lee MH, Yu Y, Chen MI, Goh EH, et al Knowledge, attitudes and practices towards antibiotic use in upper respiratory tract infections among patients seeking primary health care in Singapore. BMC Fam Pract. 2016;17(1):148 doi: 10.1186/s12875-016-0547-3 27809770 10.1186/s12875-016-0547-3 PMC5094024

[24] Bradley SF. Principles of Antimicrobial Therapy in Older Adults. Clin Geriatr Med. 2016;32(3):443–57. doi: 10.1016/j.cger.2016.02.009 27394016 10.1016/j.cger.2016.02.009

[25] Afekouh H, Baune P, Abbas R, De Falvelly D, Guermah F, Haber N. Antibiotic prescription evaluation in the rehabilitation ward of a geriatric hospital. Med Mal Infect. 2015;45(11–12):427–35. doi: 10.1016/j.medmal.2015.09.010 26573777 10.1016/j.medmal.2015.09.010

[26] Malo-Fumanal S, Rabanaque-Hernández MJ, Feja-Solana C, Lallana-Alvarez MJ, Armesto-Gómez J, Bjerrum L. Differences in outpatient antibiotic use between a Spanish region and a Nordic country. Enferm Infecc Microbiol Clin. 2014;32(7):412–7. doi: 10.1016/j.eimc.2013.10.002 24262316 10.1016/j.eimc.2013.10.002

[27] Barroso C, Abásolo I, Cáceres JJ. Health inequalities by socioeconomic characteristics in Spain: the economic crisis effect. Int J Equity Health. 2016;15:62 doi: 10.1186/s12939-016-0346-4 27067675 10.1186/s12939-016-0346-4 PMC4827195

[28] da Costa FA, Teixeira I, Duarte-Ramos F, Proença L, Pedro AR, Furtado C, et al Effects of economic recession on elderly patients' perceptions of access to health care and medicines in Portugal. Int J Clin Pharm. 2017;39(1):104–112. doi: 10.1007/s11096-016-0405-3 27933488 10.1007/s11096-016-0405-3

[29] Malo S, José Rabanaque M, Feja C, Lallana MJ, Aguilar I, Bjerrum L. High antibiotic consumption: a characterization of heavy users in Spain. Basic Clin Pharmacol Toxicol. 2014;115(3):231–6. doi: 10.1111/bcpt.12211 24517562 10.1111/bcpt.12211

[30] Knox K. Women should be able to get antibiotics for urinary tract infection without a prescription. BMJ. 2015;351:h3441 doi: 10.1136/bmj.h3441 26173946 10.1136/bmj.h3441

[31] Gaardbo Kuhn K, Hammerum AM, Jensen US. The association between demographic factors and increased antibiotic consumption in Denmark 2001 to 2010. Scand J Infect Dis. 2014;46(8):599–604. doi: 10.3109/00365548.2014.912347 24832851 10.3109/00365548.2014.912347

[32] Haeseker MB, Dukers-Muijrers NH, Hoebe CJ, Bruggeman CA, Cals JW, Verbon A. Trends in antibiotic prescribing in adults in Dutch general practice. PLoS One. 2012;7(12):e51860 doi: 10.1371/journal.pone.0051860 23251643 10.1371/journal.pone.0051860 PMC3520879

[33] Nessighaoui H, Géniaux H, Dantoine T, Laroche ML. Medicines and frailty in older people. Towards a new nosological entity: A pharmacological frailty? Therapie. 2016; 71(3):275–9. doi: 10.1016/j.therap.2016.02.021 27235650 10.1016/j.therap.2016.02.021

[34] Cooper JA, Cadogan CA, Patterson SM, Kerse N, Bradley MC, Ryan C, et al Interventions to improve the appropriate use of polypharmacy in older people: a Cochrane systematic review. BMJ Open. 2015;5(12):e009235 doi: 10.1136/bmjopen-2015-009235 26656020 10.1136/bmjopen-2015-009235 PMC4679890

[35] Marra F, Mak S, Chong M, Patrick DM. The relationship among antibiotic consumption, socioeconomic factors and climatic conditions. Can J Infect Dis Med Microbiol 2010; 21(3): 99–106. 10.1155/2010/965268 PMC2951810 21886643

[36] Kliemann BS, Levin AS, Moura ML, Boszczowski I, Lewis JJ. Socioeconomic Determinants of Antibiotic Consumption in the State of São Paulo, Brazil: The Effect of Restricting Over-The-Counter Sales. PLoS One. 2016;11(12):e0167885 doi: 10.1371/journal.pone.0167885 27941993 10.1371/journal.pone.0167885 PMC5152856

[37] Touboul-Lundgren P, Jensen S, Drai J, Lindbæk M. Identification of cultural determinants of antibiotic use cited in primary care in Europe: a mixed research synthesis study of integrated design "Culture is all around us". BMC Public Health. 2015;15:908 doi: 10.1186/s12889-015-2254-8 26381376 10.1186/s12889-015-2254-8 PMC4574721

[38] Beckett CL, Harbarth S, Huttner B. Special considerations of antibiotic prescription in the geriatric population. Clin Microbiol Infect. 2015;21(1):3–9. doi: 10.1016/j.cmi.2014.08.018 25636920 10.1016/j.cmi.2014.08.018

[39] Cardoso T, Ribeiro O, Aragão IC, Costa-Pereira A, Sarmento AE. Additional risk factors for infection by multidrug-resistant pathogens in healthcare-associated infection: a large cohort study. BMC Infect Dis. 2012;12:375 doi: 10.1186/1471-2334-12-375 23267668 10.1186/1471-2334-12-375 PMC3566942

[40] Coenen S, Ferech M, Haaijer-Ruskamp FM, Butler CC, Vander Stichele RH, Verheij TJ, et al European Surveillance of Antimicrobial Consumption (ESAC): quality indicators for outpatient antibiotic use in Europe. Qual Saf Health Care. 2007;16(6):440–5. doi: 10.1136/qshc.2006.021121 18055888 10.1136/qshc.2006.021121 PMC2653179

