# Supplementary material for: Time trends in antibiotic consumption in the elderly: Ten-year follow-up of the Spanish National Health Survey and the European Health Interview Survey for Spain (2003–2014)
Source: PLoS One. 2017 Nov 29;12(11):e0185869. doi: 10.1371/journal.pone.0185869 (PMC5706724; doi:10.1371/journal.pone.0185869)
Supplement: S1 Table — (DOC) [file pone.0185869.s001.doc]

**Table 1 in S1 Text**

**Table 1. Prevalence of consumption of prescription antibiotics in Men aged ≥65 years in Spain, according to sociodemographic variables, lifestyle; health profile and healthcare resources. Spanish National Health Survey (SNHS) 2003-2006, 2012 and European Health Interview Survey (EHIS) 2009 and 2014 for Spain.**

| **Prevalence in elderly men** | | **2003** | **2006** | **2009** | **2012** | **2014** | **Total** | **p-trend** |
| --- | --- | --- | --- | --- | --- | --- | --- | --- |
|  |  | **N (%)** | **N (%)** | **N (%)** | **N (%)** | **N (%)** | **N (%)** |  |
|  |  | 71(3.08) | 154(5.47) | 81(3.48) | 113(5.08) | 147(5.6) | 566(4.6) | 0.002 |
| **Age ^d^** | 65-74 | 39(2.97) | 78(5.35) | 39(3.23) | 41(3.59) | 70(5.07) | 267(4.11) | 0.004 |
|  | 75-84 | 25(3.08) | 60(5.37) | 34(3.82) | 52(6.24) | 63(6.76) | 234(5.1) | 0.233 |
|  | ≥ 85 | 7(3.98) | 16(6.75) | 8(3.45) | 20(8.13) | 14(4.49) | 65(5.4) | 0.831 |
| **Educational level** | No formal education | 28(3.77) | 54(6.32) | 30(3.25) | 40(5.24) | 42(5.54) | 194(4.8) | 0.207 |
|  | Primary education | 30(2.58) | 63(4.7) | 26(3.27) | 59(5.79) | 73(5.84) | 251(4.51) | 0.033 |
|  | Secondary education | 13(3.25) | 36(5.94) | 25(4.13) | 14(3.17) | 32(5.19) | 120(4.5) | 0.013 |
| **Marital status ^e^** | Single/Divorced/widow | 23(3.55) | 52(5.92) | 29(4.23) | 35(5.04) | 30(3.8) | 169(4.57) | 0.383 |
|  | Married or living together | 48(2.9) | 102(5.27) | 52(3.16) | 78(5.1) | 117(6.38) | 397(4.62) | 0.000 |
| **Monthly income ^b^** | < 970 € | 33(2.8) | 86(6.32) | 37(3.55) | 47(5.49) | 43(5.58) | 246(4.72) | 0.000 |
|  | 970–1400 € | 13(3.81) | 19(3.43) | 19(4.42) | 31(6.16) | 40(6.5) | 122(4.99) | 0.530 |
|  | More than 1400 € | 11(3.46) | 39(6.12) | 15(3.18) | 16(4.15) | 39(5.49) | 120(4.76) | 0.295 |
| **Alcohol consumption** | No | 33(3.87) | 138(5.35) | 22(3) | 99(4.96) | 58(7.56) | 350(5.05) | 0.004 |
|  | Yes | 38(2.62) | 16(6.84) | 59(3.69) | 14(6.11) | 89(4.79) | 216(4.02) | 0.009 |
| **Smoking habit** | No | 63(3.34) | 138(5.84) | 74(3.73) | 100(5.24) | 135(5.94) | 510(4.9) | 0.007 |
|  | Yes | 8(1.91) | 16(3.55) | 7(2.03) | 13(4.15) | 12(3.4) | 56(2.98) | 0.367 |
| **Physical activity ^b,d,e^** | No | 45(3.57) | 62(7.3) | 61(3.73) | 71(7.1) | 84(8.36) | 323(5.62) | 0.000 |
|  | Yes | 26(2.49) | 92(4.68) | 20(2.89) | 42(3.43) | 63(3.89) | 243(3.71) | 0.008 |
| **Body Mass Index Kg/sq.m ^d^** | <25 Normal | 15(2.29) | 39(5.58) | 22(3.65) | 14(2.87) | 38(5.4) | 128(4.07) | 0.028 |
|  | 25-29 Overweight | 39(3.36) | 62(4.94) | 35(3.3) | 52(5.21) | 74(5.9) | 262(4.57) | 0.010 |
|  | ≥ 30 Obesity | 14(3.33) | 39(7.69) | 20(4.56) | 28(6.25) | 29(5.5) | 130(5.55) | 0.231 |
| **Number of chronic condition ^a,b,c,d,e^** | None | 5(1.13) | 9(4.29) | 9(2.47) | 2(1.09) | 2(0.9) | 27(1.9) | 0.135 |
|  | 1-2 | 22(2) | 31(3.65) | 28(2.71) | 10(1.55) | 23(2.88) | 114(2.57) | 0.112 |
|  | ≥ 3 | 44(5.8) | 114(6.5) | 44(4.72) | 101(7.26) | 122(7.61) | 425(6.6) | 0.320 |
| **Medical consultation ^a,b,c,d,e^** | No | 4(0.5) | 47(3.86) | 24(1.97) | 21(1.97) | 38(2.73) | 134(2.35) | 0.008 |
|  | Yes | 67(4.47) | 107(6.71) | 57(5.13) | 92(7.97) | 109(8.84) | 432(6.55) | 0.038 |
| **Hospitalization in preceding 12 months ^a,b,c,d,e^** | No | 49(2.54) | 106(4.51) | 51(2.68) | 82(4.34) | 84(3.79) | 372(3.62) | 0.048 |
|  | Yes | 22(5.87) | 48(10.34) | 30(6.98) | 31(9.28) | 63(15.44) | 194(9.65) | 0.000 |
| **Emergency visit in preceding 12 months ^a,b,d,e^** | No | 36(2.14) | 72(3.55) | 0(0) | 51(3.06) | 74(3.78) | 233(3.17) | 0.279 |
|  | Yes | 35(5.65) | 82(10.46) | 0(0) | 62(11.19) | 73(10.96) | 252(9.6) | 0.026 |
| **Number of non-antibiotic drug ^a,b,c,d,e^** | None | 6(1.59) | 15(4.31) | 10(2.22) | 4(0.7) | 5(1.19) | 40(1.85) | 0.012 |
|  | 1-3 | 37(2.23) | 65(3.56) | 41(2.93) | 46(3.95) | 53(3.52) | 242(3.21) | 0.287 |
|  | ≥4 | 28(10.41) | 74(11.54) | 30(6.25) | 63(12.91) | 89(12.71) | 284(11.02) | 0.073 |
| **Alternative medicines** | No | 70(3.13) | 149(5.45) | 0(0) | 111(5.08) | 143(5.55) | 473(4.86) | 0.011 |
|  | Yes | 1(1.54) | 5(6.25) | 0(0) | 2(5.13) | 4(8.16) | 12(5.15) | 0.904 |
| **Daily Living Activities ^a,b,c,d,e^** | No | 50(2.48) | 112(4.6) | 55(2.95) | 69(3.77) | 97(4.45) | 383(3.71) | 0.005 |
|  | Yes | 21(7.39) | 42(11.08) | 26(5.62) | 44(11.14) | 50(11.31) | 183(9.32) | 0.082 |
| **Instrumental Daily Living Activities ^a,b,c,d,e^** | No | 44(2.34) | 80(3.86) | 47(2.74) | 62(3.73) | 96(4.7) | 329(3.51) | 0.075 |
|  | Yes | 27(6.32) | 74(10.01) | 34(5.51) | 51(9.09) | 51(8.76) | 237(8.1) | 0.061 |
| **Self-assessment of health status ^a,b,c,d,e^** | Very good / Good | 17(1.57) | 43(3.23) | 30(2.73) | 26(2.26) | 29(2.17) | 145(2.42) | 0.107 |
|  | Fair / Poor / Very poor | 54(4.43) | 111(7.48) | 51(4.15) | 87(8.1) | 118(9.15) | 421(6.69) | 0.000 |

This is the S1 Table 1 legend.

^a^ Statistically significant differences (p < 0.05) SNHS 2003; ^b^ Statistically significant differences (p < 0.05) SNHS 2006; ^c^ Statistically significant differences (p < 0.05) EHIS 2009; ^d^ Statistically significant differences (p < 0.05) SNHS 2012; ^e^ Statistically significant differences (p < 0.05) EHIS 2014
