# Supplementary material for: Time trends in antibiotic consumption in the elderly: Ten-year follow-up of the Spanish National Health Survey and the European Health Interview Survey for Spain (2003–2014)
Source: PLoS One. 2017 Nov 29;12(11):e0185869. doi: 10.1371/journal.pone.0185869 (PMC5706724; doi:10.1371/journal.pone.0185869)
Supplement: S2 Table — (DOC) [file pone.0185869.s002.doc]

**Table 2 in S2 Text**

**Table 2. Prevalence of consumption of prescription antibiotics in Women aged ≥65 years in Spain, according to sociodemographic variables, lifestyle; health profile and healthcare resources. Spanish National Health Survey (SNHS) 2003-2006, 2012 and European Health Interview Survey (EHIS) 2009 and 2014) for Spain.**

| **Prevalence in elderly women** | | **2003** | **2006** | **2009** | **2012** | **2014** | **Total** | **p-trend** |
| --- | --- | --- | --- | --- | --- | --- | --- | --- |
|  |  | **N (%)** | **N (%)** | **N (%)** | **N (%)** | **N (%)** | **N (%)** |  |
|  |  | 141(3.68) | 272(5.42) | 161(4.36) | 185(5.04) | 242(6.21) | 1001(4.98) | 0.001 |
| **Age** | **65-74** | 74(3.69) | 134(5.42) | 85(5.18) | 80(5.04) | 103(5.9) | 476(5.04) | 0.018 |
|  | **75-84** | 47(3.28) | 112(5.49) | 56(3.65) | 80(5.28) | 97(6.51) | 392(4.89) | 0.002 |
|  | **≥ 85** | 20(5.09) | 26(5.1) | 20(3.85) | 25(4.39) | 42(6.36) | 133(5.02) | 0.268 |
| **Educational level ^a,c^** | **No formal education** | 69(4.62) | 118(5.77) | 90(4.78) | 88(5.67) | 101(6.86) | 466(5.52) | 0.192 |
|  | **Primary education** | 57(2.97) | 118(4.99) | 39(3.08) | 75(4.39) | 114(5.82) | 403(4.37) | 0.000 |
|  | **Secondary education** | 15(3.58) | 35(5.87) | 32(5.9) | 22(5.34) | 27(5.81) | 131(5.38) | 0.220 |
| **Marital status** | **Single/Divorced/widow** | 84(3.54) | 163(5.47) | 97(4.11) | 109(4.76) | 147(5.95) | 600(4.81) | 0.031 |
|  | **Married or living together** | 57(3.9) | 109(5.34) | 64(4.79) | 76(5.49) | 95(6.67) | 401(5.24) | 0.028 |
| **Monthly income** | **< 970 €** | 93(4.04) | 164(5.76) | 81(4.28) | 89(4.83) | 106(6.11) | 533(5.02) | 0.060 |
|  | **970–1400 €** | 14(3.63) | 42(5.48) | 23(4.62) | 38(6.63) | 44(6.29) | 161(5.51) | 0.486 |
|  | **≥ 1400 €** | 19(4.62) | 39(5) | 34(4.78) | 16(4.27) | 46(6.67) | 154(5.19) | 0.445 |
| **Alcohol consumption ^e^** | **No** | 104(3.78) | 251(5.57) | 98(4.13) | 171(5.21) | 169(6.83) | 793(5.15) | 0.000 |
|  | **Yes** | 37(3.43) | 21(4.09) | 63(4.75) | 14(3.61) | 73(5.13) | 208(4.4) | 0.087 |
| **Smoking habit** | **No** | 138(3.66) | 269(5.49) | 154(4.29) | 177(4.97) | 230(6.15) | 968(4.95) | 0.000 |
|  | **Yes** | 3(4.84) | 3(2.38) | 7(6.42) | 8(6.96) | 12(7.69) | 33(5.81) | 0.334 |
| **Physical activity ^a,d,e^** | **No** | 104(4.23) | 126(5.61) | 104(4.18) | 120(5.73) | 146(7.16) | 600(5.3) | 0.003 |
|  | **Yes** | 37(2.69) | 146(5.26) | 57(4.72) | 65(4.12) | 96(5.17) | 401(4.56) | 0.007 |
| **Body Mass Index Kg/sq.m ^a,d^** | **<25 Normal** | 34(2.58) | 56(5.08) | 46(4.59) | 34(3.66) | 73(6.11) | 243(4.38) | 0.003 |
|  | **25-29 Overweight** | 60(3.91) | 87(5.8) | 47(3.79) | 60(5.43) | 77(5.65) | 331(4.91) | 0.046 |
|  | **≥ 30 Obesity** | 40(4.86) | 59(6.7) | 42(5.56) | 50(7.04) | 66(7.84) | 257(6.41) | 0.771 |
| **Number of chronic condition ^a,b,c,d,e^** | **None** | 10(2.27) | 4(2.17) | 3(0.95) | 5(3.05) | 2(1.01) | 24(1.84) | 0.148 |
|  | **1-2** | 23(1.49) | 22(2.22) | 31(2.72) | 13(1.78) | 24(2.84) | 113(2.15) | 0.037 |
|  | **≥ 3** | 108(5.86) | 246(6.4) | 127(5.67) | 167(6.01) | 216(7.57) | 864(6.37) | 0.273 |
| **Medical consultation ^a,b,c,d,e^** | **No** | 3(0.27) | 68(3.41) | 48(2.77) | 39(2.45) | 59(2.86) | 217(2.55) | 0.000 |
|  | **Yes** | 138(5.11) | 204(6.74) | 113(5.75) | 146(7.02) | 183(9.98) | 784(6.75) | 0.000 |
| **Hospitalization in preceding 12 months ^a,b,c,d,e^** | **No** | 98(2.99) | 202(4.67) | 125(3.97) | 126(3.91) | 174(5.15) | 725(4.18) | 0.000 |
|  | **Yes** | 43(7.72) | 70(10.09) | 36(6.61) | 59(13) | 68(13.18) | 276(9.98) | 0.004 |
| **Emergency visit in preceding 12 months ^a,b,d,e^** | **No** | 67(2.56) | 131(3.77) | 0(0) | 85(3.29) | 102(3.83) | 385(3.4) | 0.030 |
|  | **Yes** | 74(6.09) | 141(9.11) | 0(0) | 100(9.2) | 140(11.36) | 455(8.95) | 0.000 |
| **Number of non-antibiotic drug ^a,b,c,d,e^** | **None** | 9(2.42) | 11(3.44) | 3(0.68) | 2(0.36) | 3(0.88) | 28(1.38) | 0.006 |
|  | **1-3** | 60(2.31) | 85(3.07) | 49(2.6) | 48(2.75) | 57(2.96) | 299(2.74) | 0.668 |
|  | **≥4** | 72(8.32) | 176(9.09) | 109(7.97) | 135(9.88) | 182(11.16) | 674(9.4) | 0.206 |
| **Alternative medicines ^b^** | **No** | 130(3.63) | 242(5.12) | 0(0) | 174(4.93) | 226(6.06) | 772(4.96) | 0.000 |
|  | **Yes** | 11(4.51) | 30(10.17) | 0(0) | 11(7.64) | 16(9.76) | 68(8.03) | 0.132 |
| **Daily Living Activities ^a,b,c,d,e^** | **No** | 89(2.94) | 178(4.58) | 85(3.45) | 99(3.92) | 128(4.58) | 579(3.94) | 0.007 |
|  | **Yes** | 52(6.52) | 94(8.26) | 76(6.17) | 86(7.48) | 114(10.35) | 422(7.79) | 0.092 |
| **Instrumental Daily Living Activities ^a,b,c,d,e^** | **No** | 79(2.79) | 153(4.41) | 101(3.63) | 93(3.89) | 94(4.16) | 520(3.79) | 0.025 |
|  | **Yes** | 62(6.19) | 119(7.67) | 60(6.56) | 92(7.18) | 148(9.06) | 481(7.53) | 0.217 |
| **Self-assessment of health status ^a,b,c,d,e^** | **Very good / Good** | 28(2.05) | 54(3.21) | 31(2.5) | 36(2.54) | 49(3.23) | 198(2.74) | 0.285 |
|  | **Fair / Poor / Very poor** | 113(4.59) | 218(6.52) | 130(5.29) | 149(6.6) | 193(8.11) | 803(6.23) | 0.001 |

This is the S2 Table 2 legend.

^a^ Statistically significant differences (p < 0.05) SNHS 2003; ^b^ Statistically significant differences (p < 0.05) SNHS 2006; ^c^ Statistically significant differences (p < 0.05) EHIS 2009; ^d^ Statistically significant differences (p < 0.05) SNHS 2012; ^e^ Statistically significant differences (p < 0.05) EHIS 2014
